# Aquaporin-Containing Proteopolymersomes in Polyelectrolyte Multilayer Membranes

**DOI:** 10.3390/membranes10050103

**Published:** 2020-05-18

**Authors:** Dennis M. Reurink, Fei Du, Radosław Górecki, Hendrik D.W. Roesink, Wiebe M. de Vos

**Affiliations:** 1 Membrane Science & Technology, University of Twente, MESA+ Institute for Nanotechnology, P.O. Box 217, 7500 AE Enschede, The Netherlands; d.m.reurink@utwente.nl (D.M.R.); feidu95@gmail.com (F.D.); h.d.w.roesink@utwente.nl (H.D.W.R.); 2 Department of Environmental Engineering, Technical University of Denmark, Bygningstorvet 115, 2800 Kongens Lyngby, Denmark; rgo@aquaporin.dk; 3 Aquaporin A/S, Nymøllevej 78, 2800 Kongens Lyngby, Denmark

**Keywords:** biomimetic membrane, nanofiltration, water, layer by layer

## Abstract

The field of membranes saw huge developments in the last decades with the introduction of both polyelectrolyte multilayer (PEM)-based membranes and biomimetic membranes. In this work, we combine these two promising systems and demonstrate that proteopolymersomes (PP+) with the incorporated aquaporin protein can be distributed in a controlled fashion using PEMs, even on the inner surface of a hollow fiber membrane. In this way, various proteopolymersome multilayers (PPMs) are fabricated using PP+ as the positively charged species in combination with the polyanions poly(styrene 4-sulfonate) (PSS) and poly(acrylic acid) (PAA). It is shown by reflectometry through alternately adsorbing the polyanions and PP+ that, for both PAA and PSS, a good layer growth is possible. However, when the multilayers are imaged by SEM, the PAA-based PPMs show dewetting, whereas vesicular structures can only be clearly observed in and on the PSS-based PPMs. In addition, membrane permeability decreases upon coating the PPMs to 2.6 L∙m^−2^∙h^−1^∙bar^−1^ for PAA/PP+ and 7.7 L∙m^−2^∙h^−1^∙bar^−1^ for PSS/PP+. Salt retentions show that PAA/PP+ layers are defective (salt retentions <10% and high molecular weight cut-off (MWCO)), in line with the observed dewetting behavior, while PPMs based on PSS show 80% MgSO_4_ retention in combination with a low MWCO. The PSS/PP+ membranes show a Donnan-exclusion behavior with moderate MgCl_2_ retention (50%–55%) and high Na_2_SO_4_ retention (85%–90%) indicating a high amount of negative charge present within the PPMs. The corresponding PEMs, on the other hand, are predominately positively charged with MgCl_2_ retention of 97%–98% and Na_2_SO_4_ retention of 57%–80%. This means that the charge inside the multilayer and, thus, its separation behavior can be changed when PP+ is used instead of a polycation. When comparing the PPM membranes to the literature, similar performances are observed with other biomimetic membranes that are not based on interfacial polymerization, but these are the only ones prepared using a desired hollow fiber geometry. Combining PEMs and biomimetic approaches can, thus, lead to relevant membranes, especially adding to the versatility of both systems.

## 1. Introduction

The field of membranes saw huge developments in the last decades with the arrival of novel promising membranes like polyelectrolyte multilayer (PEM)-based membranes and biomimetic membranes. Both PEM-based membranes and biomimetic membranes were investigated in a variety of studies, and both are already commercially produced and applied [1]. 

PEMs are fabricated using two oppositely charged polyelectrolytes that are adsorbed alternately on top of a charged surface [2]. Using PEMs, membranes can be fabricated by applying the PEMs on top of a porous support membrane. Using this coating method, PEM membranes were studied for numerous applications regarding water purification, e.g., for reverse osmosis (RO) [3], forward osmosis (FO) [4], and nanofiltration (NF) [5] processes. However, when looking at these processes in more detail, PEM membranes are not really competitive as RO membranes, as they can only be made selective enough for RO at a huge cost of permeability [3,5]. Due to the low permeability of PEM-based RO membranes, these types of membranes currently do not outperform polyamide-based RO membranes. However, their relatively high permeability and the tunability of their selectivity make PEMs are ideal candidates for NF membranes. In addition, PEMs can be easily coated on the inside of hollow fibers, allowing manufacturers to produces modules with a higher aspect ratio and without spacers which are prone to fouling [6]. PEM membranes can, furthermore, be easily cleaned due to their high chemical and physical stability, especially in comparison to polyamide-based membranes [7].

Biomimetic membranes are a collection of membranes which are inspired by biological membranes present in the walls of cells [8]. One type of biomimetic membranes is based on the aquaporin protein which acts as a selective water channel and allows water molecules to pass up to one billion molecules per second per channel [9]. The water molecules pass the ultra-narrow hydrophobic channel by moving in a single file, allowing for the passage of water while retaining all other solutes [10]. This high selectivity toward water is due to the size restriction, charge repulsion [11], and the specific dipole orientation of water [12] within the aquaporin water channel. For the reasons above, the aquaporin water channel is very promising since, in RO and NF operations, high selectivities are needed which could be obtained with these channels on the condition that water is only permeating through the aquaporin channels [13]. Moreover, due to the high throughput of water molecules, the permeability of both NF and RO membranes can potentially be increased [14].

In order to retain the structure and function of the aquaporin water channel, the protein has to be reconstituted into an phospholipid bilayer [15] or an amphiphilic block copolymer bilayer [16,17]. This can be achieved by embedding the aquaporin proteins into vesicles, i.e., liposomes or polymersomes. Of the two, polymersomes show a higher physical and chemical stability [18] and a tunable thickness and permeability [19]. To make membranes that contain the aquaporin protein, two approaches were studied, namely, supported bilayers [18] and vesicle-embedded membranes [20,21]. Although supported bilayers show higher permeabilities, they are prone to mechanical damage and degradation and are highly difficult to produce in a defect-free manner. For this reason, vesicle-embedded membranes are used in this study since the vesicles can easily be incorporated into an existing layer that supports the vesicles and prevents defects [14]. Moreover, Górecki et al. showed that aquaporin-containing proteopolymersomes were successfully added to polyamide layers, where they contributed to an improved membrane performance [22].

Looking at both approaches, combining biomimetic membranes with PEMs is interesting because of the high selectivity and permeability of aquaporin water channels and the tunability of PEMs. Moreover, by relying on electrostatic interactions with polyelectrolytes (PEs), aquaporin-containing proteoliposomes or proteopolymersomes can be easily distributed to form a layer with a high packing density of vesicles. Previous studies already reported how to distribute aquaporin-containing vesicles using magnetic particles inside the vesicles [23], by interaction with PEs [24], by mixing them in the aqueous phase during interfacial polymerization [20], or by incubating the substrate [21]. In this way, these studies controlled the vesicle distribution on the surface and constructed membranes with visible vesicles on the surface. However, to assess the vesicle distribution, simple methods are missing in order to quantify the number of vesicles within the active layer of the membrane. Therefore, in this study, aquaporin-containing proteopolymersomes are adsorbed in a controlled manner on model surfaces to study the growth of vesicles containing multilayers and translate this knowledge to allow controlled layer formation on the inner surface of hollow fiber membranes. There were published studies and patents reporting proteoliposomes/proteopolymersomes on the inside of a hollow fiber configuration; however, this was all performed using interfacial polymerization (IP) [25,26,27,28]. The IP fabrication method is hard to control on a hollow fiber geometry; therefore, a more straightforward method to produce hollow fiber-based biomimetic membranes would be very beneficial. 

Therefore, in this study, we propose a simple method to form hollow fiber proteopolymersome multilayer (PPM) membranes based on sequential dip-coating of PEs and proteopolymersomes (PP+). The PPMs are based on three different multilayers varying in interaction between weak and strong PEs: poly(acrylic acid) (PAA)/poly(allyl amine) (PAH) [29], poly(styrene 4-sulfonate) (PSS)/PAH [30], and PSS/poly(diallyl dimethyl ammonium chloride) (PDADMAC) [31]. PAA/PAH multilayers consist of two weak PEs resulting in a dense layer (low molecular weight cut-off (MWCO)) with low permeability, but also low salt retention [32]. Changing the polyanion from a weak to a strong PE, creating a PSS/PAH multilayer, results in a totally different multilayer with both high permeability and high salt retention [33]. When the polycation is subsequently switched from weak to strong, a PSS/PDADMAC multilayer is fabricated with high permeability and moderate salt retention, but with an excellent chemical stability due to the quaternary amine of PDADMAC [7]. The polycations in the mentioned PEMs are simply substituted by the PP+ to build a PPM, fabricating in essence two different PPMs: a PAA/PP+ and a PSS/PP+ PPM. Here, the fabricated PPMs are compared with the traditional PEMs to obtain a deeper understanding of how the PP+ affect the composition of the multilayer and, therefore, how they affect the performance of the membrane. It is shown by studying the PPM growth on model surfaces with reflectometry that PPMs can be grown in combination with different polyanions. On the inside of hollow fiber membranes, using SEM and membrane performance measurements, it is further shown that the PPM layers can be fabricated and used as an active layer. Subsequently, the nanofiltration performance of the membranes is tested and compared to the original PEMs without any PP+ and to values reported in the literature.

## 2. Materials and Methods

### 2.1. Materials

Poly(diallyldimethylammonium chloride) (PDADMAC, molecular weight (M_W_) = 200,000–350,000 g∙mol^−1^, 20 wt.% in water), poly(sodium 4–styrenesulfonate) (PSS, M_W_ = 200,000 g∙mol^−1^, 30 wt.% in water), and poly(acrylic acid) (PAA, M_W_ = 250,000 g∙mol^−1^, 35 wt.% in water) were purchased from Sigma-Aldrich, and poly(allylamine hydrochloric acid) (PAH, M_W_ = 150,000 g∙mol^−1^, 40 wt.% in water) was obtained from Nittobo Medical, Tokyo, Japan. Sodium chloride was purchased from Akzo Nobel, Hengelo, The Netherlands, magnesium chloride and magnesium sulfate were purchased from Boom B.V. (Meppel, The Netherlands), and sodium sulfate was purchased from Sigma-Aldrich (Schnelldorf, Germany). 

Proteopolymersomes (PP+) were obtained from Aquaporin A/S, Denmark. PP+ consisted of a 1.12 mg/mL mixture of poly(2-methyl-2-oxazoline)–*block*–poly(dimethylsiloxane) (PMOXA–PDMS) diblock copolymer, poly(2-methyl-2-oxazoline)–*block*–poly(dimethylsiloxane)–*block*–poly(2-methyl-2-oxazoline) (PMOXA–PDMS–PMOXA) triblock copolymer, and bis(3-aminopropyl)-terminated poly(dimethylsiloxane) (A-PDMS) in phosphate-buffered saline (PBS) with 0.05% lauryldimethylamine *N*-oxide (LDAO), 0.5% Kolliphor® HS 15 (KHS), and 5 mg/L aquaporin Z protein. They were prepared via bulk hydration self-assembly, according to procedures described by M. Spulber et al. [34] and in a study by Górecki et al. [22] The average particle size of PP+ was reported to be 150 ± 1 nm with total particle concentration of 3.26 × 10^11^ ± 7.08 × 10^9^ particles/mL, and a neutral to slightly positive ζ-potential (+0.9 mV) due to the A-PDMS.

Obtained PP+ were filtered by a 0.4-μm Whatman filter prior to use. To check the PP+ solution quality, the filtered solution was measured by dynamic light scattering (DLS) Zetasizer Nano Zs (Malvern, United Kingdom); if a >85% intensity peak at 150–250 nm was observed, then the solution was recognized as good quality [22].

Tight ultrafiltration hollow fiber membranes were obtained from NX Filtration B.V. (Enschede, The Netherlands) and used as supports. The charge of the supports was positive, the inner diameter was 0.68 nm, the standard permeability was 200 L∙m^−2^∙h^−1^∙bar^−1^, and the fibers had a molecular weight cut-off of 25 kDa. The radial structure of the hollow fibers was asymmetrical inside-out, meaning that the smallest pore size was present on the inside of the fiber. Silicon wafers were obtained from WaferNet Inc. (San Jose, CA, USA).

### 2.2. Reflectometry

The build-up of polyelectrolyte multilayers (PEMs) and PP+ multilayers was measured quantitatively using optical fixed-angle reflectometry on top of a silicon wafer with a top layer of 80-nm silicon oxide. All polyelectrolyte (PE) solutions contained 0.1 g∙L^−1^ PE and 50 mM NaCl, and they were adjusted to a pH of 2 for PAH and pH of 6 for PAA, with no pH adjustment for PDADMAC and PSS. The PP+ solutions were not adjusted in terms of the pH and no additional salt was added. Rinsing solutions contained the same ionic strength as the PE solutions with no pH adjustment.

Prior to the measurement, silicon wafers were oxygen plasma-treated by a Femto plasma cleaner (Diener electronic GmbH, Ebhausen, Germany) to clean and to obtain a reproducible SiO_2_ surface chemistry. Subsequently, the wafer was put into the reflectometry flow cell while the rinsing solvent was flowing. The first layer was a layer of PAH to create a stable primer layer in order to grow the PPM. The first layer was adsorbed by flowing a PAH solution onto the silicon wafer until a stable adsorption plateau was reached. After the first layer, the rinsing solution was flowed until the adsorption was stable. Subsequently, the second layer was adsorbed by flowing either a PSS or a PAA solution. After rinsing, the PP+ solution was flowed and, by alternately adsorbing a polyanion (PSS or PAA) and the PP+ solution with rinsing steps in between, a PPM could be built. All experiments took place under ambient conditions.

Reflectometry uses monochromatic light (He–Ne laser, 628.8 nm) which is linearly polarized. The laser beam is reflected from the silicon wafer at an angle of 71°, also known as the Brewster angle. In the detector, the laser light is split into its parallel and perpendicular components. Equation (1) shows that the difference of these components divided by the initial state is proportional to the amount of mass adsorbed on top of the silicon wafer.

(1) $$\Gamma =Q\cdot \frac{\Delta S}{S_{0}}$$

In Equation (1), Γ is the amount of mass adsorption on the silicon wafer in mg∙m^−2^. ΔS is the signal difference between the parallel and perpendicular component, and S_0_ is the initial signal. Q is known as the Q-factor or sensitivity factor and depends on the refractive indices, thickness of the silicon oxide layer and adsorbed layers, angle of incidence, and the refractive index increment of the multilayer. An optical model was used to estimate the sensitivity factor of a PPM on top of a silicon wafer with 80 nm of silicon oxide. The sensitivity factors were calculated to be 24 and 25 mg∙m^−2^ for PAA/PAH and PSS/PAH multilayers, respectively. For the according PPMs, the same sensitivity factors were used in order to calculate the adsorption.

### 2.3. Scanning Electron Microscopy

Surface and cross-sections images were taken by a field-emission scanning electron microscope (JSM-7610F, JEOL, Tokyo, Japan). Prior to imaging, the samples were sputter-coated (Quorum Q150T ES, Lewes, UK) by a 5-nm-thick chromium layer. All samples were dried in a vacuum oven prior to sputter coating, and the images were taken with an accelerating voltage of 1.0 kV.

### 2.4. Transmission Electron Microscopy

Solution samples containing proteopolymersome were introduced onto the carbon grid. Subsequently, the solution on the grid was incubated for 5 min; excess solution was then drained off the bottom of the carbon grid using a filter paper. The sample was washed with deionized water and stained by adding an aqueous solution containing 1% of phosphotungstic acid. Excess solution was again drained from the bottom of the carbon grid using a filter paper. Imaging was conducted using a 200-kV JEM-2100F transmission electron microscope (JEOL, Peabody, Massachusetts, USA).

### 2.5. Membrane Performance Experiments

For hollow fiber membrane performance measurements, the coated hollow fiber membranes were potted into single-fiber modules using PE tubing with a 6-mm outer diameter. A cross-flow set-up in which hydraulic pressure is applied by a rotary vane pump (BN71B4 pump motor, Bonfiglioli, Italy; IMTI 1.5M inverter, Electroil, Italy; PA411 pump head, Fluid-o-Tech, Italy) was used to perform permeability and salt retention measurements.

The permeability was measured using demineralized water in a cross-flow set-up at an applied transmembrane pressure of 2 bars. At least 10 mL of permeate was collected before measuring the weight and time. Salt retention was measured using 5 mM NaCl, MgSO_4_, MgCl_2_, or Na_2_SO_4_ at an applied transmembrane pressure of 2 bars and a cross-flow velocity of 1 m∙s^−1^, which corresponds to a Reynolds of 675, well within the laminar flow regime. Retention was determined by measuring the feed and permeate conductivity using a WTW conductivity meter; subsequently, the retention was calculated using Equation (2). At least 20 mL of permeate was collected before the permeate conductivity was measured.

(2) $$R=\frac{\Delta C}{C_{feed}}\cdot 100\%.$$

MWCO was determined in the same conditions as the salt retention experiments, and the determination was performed by permeating a solution containing ethylene glycol (EG), diethylene glycol (DEG), and various molecular weights of poly(ethylene glycol) (PEG). The following molecular weight were used; EG62, DEG106, and PEG (200, 400, 600, 1000, 1500, and 2000 g∙moL^−1^). For each molecule, 1 g∙L^−1^ was dissolved in demineralized water.

## 3. Results and Discussion

**Figure 1:**
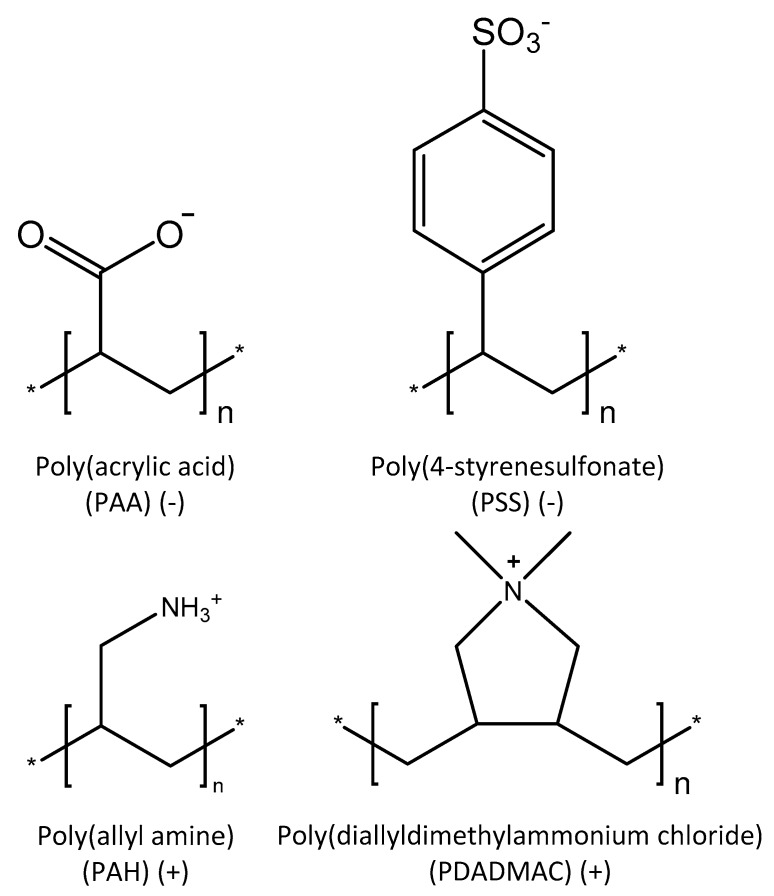
Polyelectrolytes used in this study in order to fabricate polyelectrolyte multilayers (PEMs) and proteopolymersome multilayers (PPMs).

This section is split into two separate parts: (1) the fundamental layer build-up of proteopolymersome (PP+) multilayers (PPMs), and (2) their performance as separation layers. In the fundamental part, the layer build-up of the two PPMs is studied by means of reflectometry and SEM images. In the membrane performance part, the permeability and MgSO_4_ retention of the fabricated multilayers are discussed as a function of the number of layers, while, for both systems, selected layers are also more thoroughly studied for their MWCO and salt retention (NaCl, MgCl_2_, and Na_2_SO_4_). Throughout, the PPMs are compared to traditional PEMs. For this comparison, three well-studied PEMs are used: PAA/PAH-, PSS/PAH-, and PSS/PDADMAC-based multilayers with the polyelectrolyte structures shown in Figure 1. As discussed in the introduction, these PEMs were chosen because of the different interactions between weak and strong polyelectrolytes and the resulting membrane characteristics and performances. Finally, the results obtained in this study are compared to literature results on aquaporin-based membranes.

### 3.1. Multilayer Characterization

**Figure 2:**
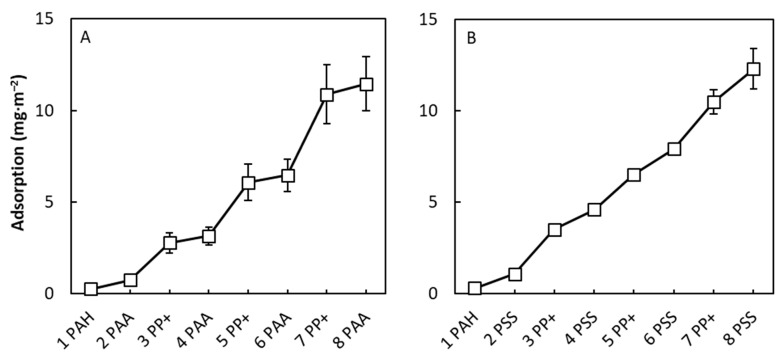
Reflectometry results on the adsorption of PPMs as function of layer number. (**A**) Poly(acrylic acid) (PAA) was used as the polyanion and proteopolymersomes (PP+) were used as the positively charged agent. (**B**) Poly(styrene 4-sulfonate) (PSS) was used as the polyanion in combination with the positively charged PP+. Error bars are standard deviations of three separate measurements. When the error bars are not visible, the error is smaller than the data point symbols.

To evaluate if multilayers can be fabricated using just PP+ and polyelectrolytes, optical fixed-angle reflectometry was used to quantitatively characterize the PPM growth using PAA and PSS as the polyanion and PP+ as the positively charged species. Similar to other multilayer studies using reflectometry, the polyelectrolyte solutions and the PP+ solutions were flowed perpendicular to the surface of the silicon wafer in an alternating fashion. The hydrodynamics in the flow cell are such that convection plays no role and that the adsorption is diffusion-limited, just as in the dip-coating processes used to coat the membranes [35]. In Figure 2, the adsorption of PPMs in combination with PAA (left figure) and PSS (right figure) is plotted as function of the number of layers. Here, we observe a steady layer growth for both polyanions, demonstrating that a multilayer can indeed be fabricated using PP+ as the positively charged species in combination with either PAA or PSS as the polyanion. Using PAA as the polyanion, as shown in Figure 2A, it is observed that the relative growth of PP+ compared to PAA is eight times larger, with a higher adsorption of 4 mg∙m^−2^ for the last PP+ layer and just 0.5 mg∙m^−2^ for the last PAA layer. Similar behavior is observed when PSS was used as the polyanion (Figure 2B); however, this occurred to a lesser extent with 2.5 mg∙m^−2^ for the last PP+ layer and 1.8 mg∙m^−2^ for the last PSS layer. This shows that that the total amount of mass of PP+ is in excess within the multilayer, indicating a high amount of PP+ inside of the layers. As an additional test, the fabrication of PPMs based on PP+ in combination with polycations was attempted. This resulted in unstable adsorption and no PPM layer growth, as expected based on the small positive charge of PP+. In summary, the incorporation of PP+ in multilayers is a relatively straightforward process in combination with the polyanions PSS and PAA.

**Figure 3:**
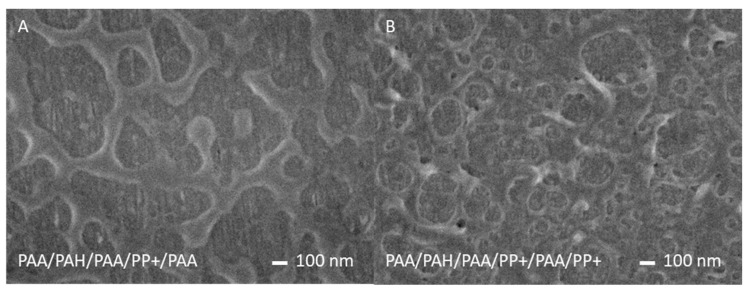
Field-emission (FE)-SEM images of PAA/PP+ multilayers on top of a [PAA/ poly(allyl amine) (PAH)]_1_PAA multilayer. (**A**) PPM terminated by PAA. (**B**) PPM terminated by PP+. All multilayers were deposited on the inside of hollow fiber membrane supports.

Subsequently, these PAA/PP+ and PSS/PP+ multilayers were fabricated on hollow fiber membrane supports and imaged by SEM. In Figure 3, PAA-based PPMs are shown, and no distinct polymersomes or vesicle shaped structures can be observed on the active surface layer of the membrane. Moreover, the clear agglomeration of polymer material looks like typical dewetting [36,37] behavior, where the polymer coatings are too mobile and do not wet the surface.

**Figure 4:**
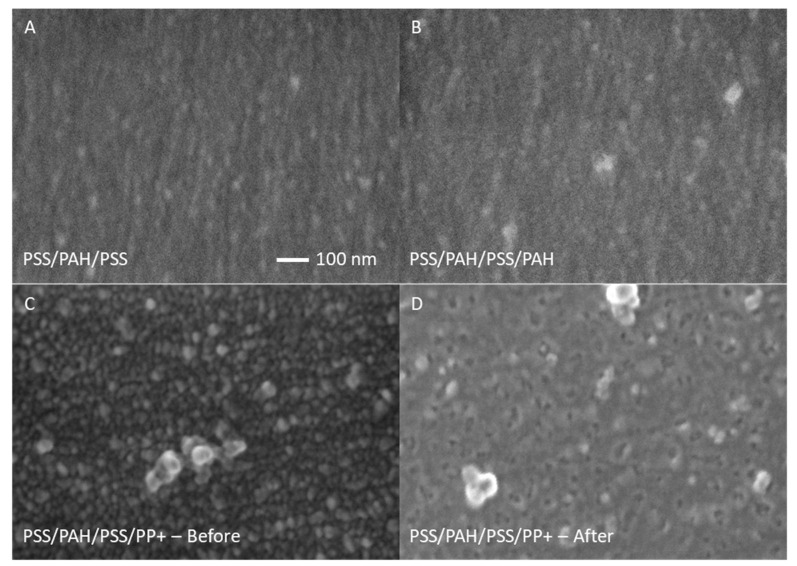
FE-SEM images of [PSS/PAH]_1_PSS and the [PSS/PAH]_2_ multilayers (**A**,**C**, respectively) and PP+-terminated multilayers before and after filtration tests (**B**,**D**, respectively). All multilayers were deposited on the inside of hollow fiber membrane supports.

For the PPMs in which PSS was used as the polyanion together with the PP+, the same procedure was followed whereby the vesicles were constructed on top of a 1.5-bilayer-thick ([PSS/PAH]_1_PSS) multilayer. In Figure 4A and C, solely the [PSS/PAH]_1_PSS and the [PSS/PAH]_2_ multilayers without PP+ are imaged and a relative smooth surface can be seen. It is observed that the PSS-terminated multilayer was smoother than the PAH-terminated multilayer, which is in accordance with roughness values reported in literature [38]. However, when having just a single layer of PP+ deposited on the surface, as shown in Figure 4B, it is observed that the surface roughness increased and that the surface was covered with small and larger components. Here, it is noticed that the smaller components outnumbered the larger components. It is expected that the larger components were the proteopolymersomes since the size should be around 150 nm according to the dynamic light scattering (DLS) measurements, as mentioned in Section 2 and the transmission electron microscopy image shown in Figure S1 (Supplementary Materials). However, the PP+ appear smaller than in the DLS measurement, most likely because they collapsed during SEM imaging due to the high vacuum used. Nevertheless, vesicular-shaped structures are clearly observed indicating that the PP+ were present on and in the active layer of the membrane for the PSS/PP+ PPMs.

It is important to check if the PPM is stable and that the PP+ are still present after membrane filtration test. In Figure 4D, the PP+-terminated surface is imaged after the membrane was exposed to membrane filtration tests. Here, it is seen that the small components were not present on the surface anymore, although the larger PP+ (vesicular structures) components were still present on the surface. This shows that, even after filtration, the PP+ were still present on the surface of the hollow fiber membrane. In Figure 4D, the pore structure below the layers seems more visible than in Figure 4C. Nevertheless, molecular weight cut-off (MWCO) measurements with PP+-terminated multilayers show that these membranes did not contain any defects. These experiments are more elaborately discussed in the next section.

### 3.2. Membrane Performance

**Figure 5:**
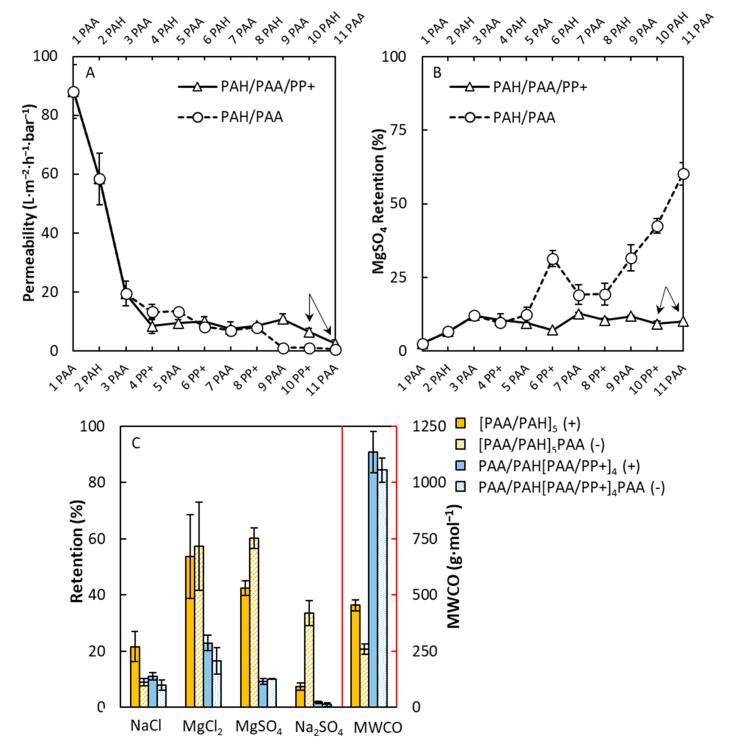
Permeability (**A**) and MgSO_4_ retention (**B**) of PAA/PP+ and PAA/PAH multilayers as a function of the number of layers. Retention of various salts (**C**) for both PAA/PP+ and PAA/PAH multilayers having 5.0 and 5.5 bilayers, respectively. Both permeability and retention measurements were performed at 2 bars of pressure and a cross-flow velocity of 1 m∙s^−1^. The arrows indicate the membranes that were used for further salt retention and molecular weight cut-off (MWCO) measurements. For the retention measurements, a concentration of 5 mM was used. Error bars are standard deviations; *n* = 5.

In this section, the membrane performance of the PPMs is assessed and compared to PEM membranes based on the same polyanions. The first multilayers tested were the PAA/PP+ and the PAA/PAH multilayers, where both the permeability and retention results are plotted in Figure 5A,B respectively. The first three layers, [PAA/PAH]_1_PAA of the PPM, were the same as the PEM to obtain a good primer layer. It is observed that the PAA/PP+ multilayer (solid line) had a higher permeability after eight layers than the PAA/PAH multilayers (dashed line). The PAA/PP+ multilayer decreased the permeability steadily with more layers and eventually obtained a permeability of 2.6 L∙m^−2^∙h^−1^∙bar^−1^, whereas the PAA/PAH multilayers had a permeability of 0.6 L∙m^−2^∙h^−1^∙bar^−1^ after 11 layers. When looking at the MgSO_4_ retention, however, it is observed that, for the PAA/PP+ multilayer, the retention stabilized already after three layers, the point after the primer layer where the PAA/PP+ multilayer started being built. The decreasing permeability in combination with the reflectometry results (Figure 2) suggests that a layer was built on the surface. However, the MgSO_4_ retentions did not show an increasing trend as seen for the control PAA/PAH multilayer. This is indicative of a defective layer, as suggested already by the SEM images in Figure 3. Furthermore, in Figure 5C, together with the retention of MgSO_4_, NaCl, MgCl_2_, and Na_2_SO_4_, the MWCO is shown for the 5.0- and 5.5-bilayer-thick PEMs and PPMs, as indicated by the arrows in Figure 5A,B. Retention of various salts was measured in order to obtain more understanding about the rejection mechanisms of all the PPMs and PEMs built. In these figures, it is clearly seen that the PAA/PP+ PPM was defective, regardless of the terminating layer, in comparison to the defect-free PAA/PAH PEM, since the overall salt retention was very low in combination with a high MWCO of around 1 kDa. The PAA/PAH PEM membranes had a relatively high retention toward MgCl_2_ and MgSO_4_, indicating that a positive charge was most dominant within these PEMs, in combination with a low 250-Da MWCO.

**Figure 6:**
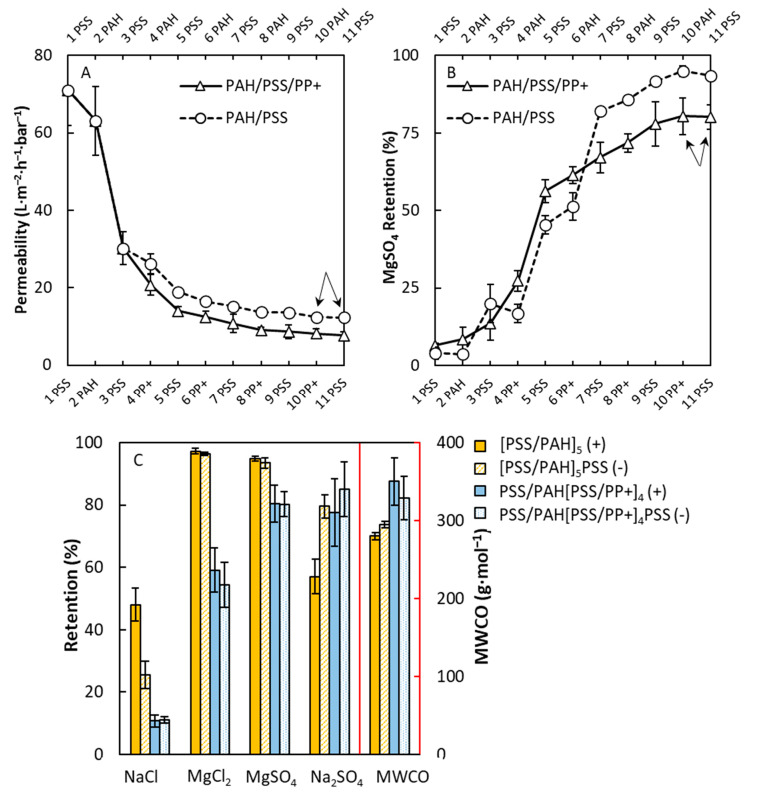
Permeability (**A**) and MgSO_4_ retention (**B**) of PSS/PP+ and PSS/PAH multilayers as a function of the number of layers. Retention of various salts (**C**) for both PAH/PSS/PP+ and PSS/PAH multilayers having 5.0 and 5.5 bilayers, respectively. Both permeability and retention measurements were performed at 2 bars of pressure and a cross-flow velocity of 1 m∙s^−1^. For the retention measurements, a concentration of 5 mM was used. The arrows indicate the membranes that were used for further salt retention and MWCO measurements. Error bars are standard deviations; *n* = 5.

The second multilayer studied was the PSS/PAH PEM multilayer and the corresponding PAH/PSS/PP+ PPM multilayer. In Figure 6A (solid line), it is seen that the permeability decreased steadily when increasing the number of layers, in line with the reflectometry results. The first two layers were Pes, and it is seen that a PSS/PP+ layer was neatly constructed after this point where the PSS/PP+ multilayer started being built. Compared to a PSS/PAH multilayer (dashed line), it is noticed that, after the primer layer ([PSS/PAH]_1_PSS) the PSS/PAH multilayer had a consistently higher permeability than the PSS/PP+ multilayer. The PSS/PP+ multilayer stabilized at a permeability of 7.7 L∙m^−2^∙h^−1^∙bar^−1^ after 11 layers, whereas the PSS/PAH multilayer stabilized at a much higher permeability of 12 L∙m^−2^∙h^−1^∙bar^−1^, indicating that the PSS/PAH multilayer was less resistant to water and, thus, more permeable than the PSS/PP+ multilayer. This could be seen as surprising, since aquaporin-containing vesicles were able to increase the permeability of membranes prepared by interfacial polymerization [20]; thus, why would a layer that contains mostly vesicles (see Figure 2B) not have an improved permeability? Here, it is important to realize that PEM coatings have such a high permeability, especially due to low thickness of the coatings (<50 nm), while the vesicles themselves are much larger in size (150–200 nm). This discrepancy in layer thickness is likely why the PEM outperformed the PPM in terms of permeability. 

When looking at the retention of MgSO_4_ as function of the layer number, shown in Figure 6B, the transition from the pore-dominating to the layer-dominating regime can be nicely observed. This transition started after four layers, and it is the point at which the multilayer started forming a layer on top of the pores instead of a layer on the wall of the pores [39]. After the complete transition from the pore-dominating to the layer-dominating regime after seven layers, it is noticed that the PSS/PAH multilayer stabilized at a MgSO_4_ retention of 94% and the PSS/PP+ multilayer stabilized at a retention of 80%. When comparing the retention toward various salts for both multilayers, as shown in Figure 6C, it is observed that the retention toward NaCl and MgSO_4_ was slightly higher for the PEM membrane than the PPM membrane. Moreover, a strong effect was seen whether the PSS/PAH multilayer was PSS- or PAH-terminated with a higher NaCl retention for the PAH-terminated multilayer and a higher Na_2_SO_4_ retention for the PSS-terminated multilayer. When comparing the MgCl_2_ and Na_2_SO_4_ retentions of the PSS/PP+ and PSS/PAH multilayers, Donnan-exclusion was seen for the PSS/PAH membranes, showing a high removal of positively charged ions and a lower removal for negative species due to an excess of PAH within the multilayer [30]. The opposite can be observed for the PSS/PP+ layers, which showed a high Na_2_SO_4_ retention but low MgCl_2_ retention, meaning that, within the PSS/PP+ layers, a negative charge was dominant due to the high charge density of PSS. This means that, by using PP+ instead of PAH, the charge balance of the multilayer could be altered, and a predominantly negatively charged multilayer could be obtained. This was likely due to the much lower charge density of the PP+ in comparison to PAH and, therefore, the negative charge of PSS became dominant.

**Figure 7:**
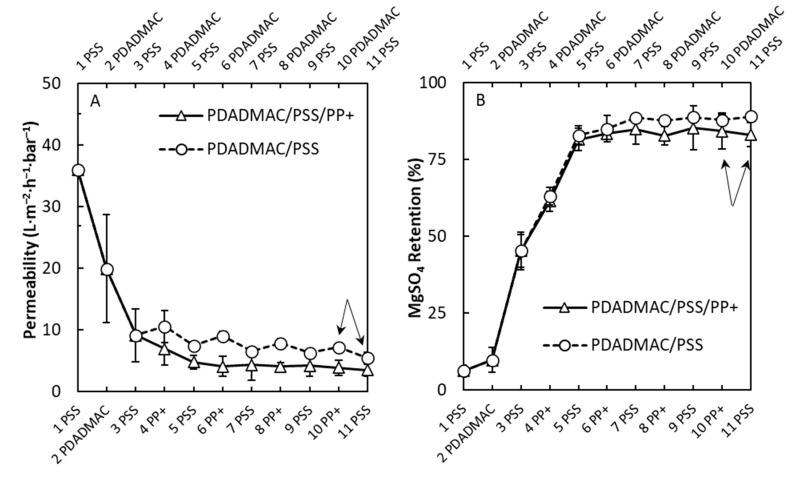

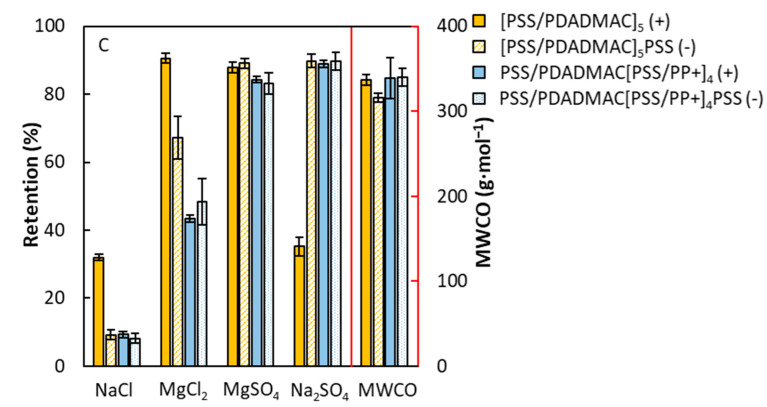
Permeability (**A**) and MgSO_4_ retention (**B**) of PSS/PP+ and PSS/poly(diallyl dimethyl ammonium chloride) (PDADMAC) multilayers as a function of the number of layers. Retention of various salts (**C**) for both PDADMAC/PSS/PP+ and PSS/PDADMAC multilayers having 5.0 and 5.5 bilayers, respectively. Both permeability and retention measurements were performed at 2 bars of pressure and a cross-flow velocity of 1 m∙s^−1^. For the retention measurements, a concentration of 5 mM was used. The arrows indicate the membranes that were used for further salt retention and MWCO measurements. Error bars are standard deviations; *n* = 5.

The third and last multilayers studied were the PSS/PDADMAC PEM and PDADMAC/PSS/PP+ PPM. In essence, the PDADMAC/PSS/PP+ and PAH/PSS/PP+ were the same PPMs based on PP+ and PSS; however, both were constructed on a different primer layer of [PAH/PSS]_1_PAH and [PDADMAC/PSS]_1_PDADMAC, respectively. The main difference between the other two PEMs studied in this article is that PSS/PDADMAC was completely built using strong PEs, whereas PSS/PAH had one weak PE and PAA/PAH had two weak PEs. In Figure 7A,B, the permeability and MgSO_4_ retention are plotted as a function of the number of layers for PSS/PP+ and PSS/PDADMAC multilayers. Here, the permeability showed a steady decrease for the PSS/PP+ multilayer after the primer layer and stabilized at 3.5 ± 1.2 L∙m^−2^∙h^−1^∙bar^−1^ after 11 layers. This shows that a PPM was built on top of the surface of the hollow fiber support membrane. The PSS/PDADMAC multilayer showed a strong odd–even effect [40] and, on average, had a higher permeability with increasing layer number of 7.2 ± 1.1 and 5.4 ± 1.2 L∙m^−2^∙h^−1^∙bar^−1^ for PDADMAC- and PSS-terminated PEMs with thicknesses of 5.0 and 5.5 bilayers, respectively. MgSO_4_ retentions stabilized at 88% ± 2% to 89% ± 1% depending on the terminated layer for the PSS/PDADMAC multilayer and stabilized at around 84% ± 7% for the PDADMAC/PSS/PP+ multilayer. When looking at Figure 7B, the retention for MgSO_4_ stabilized around the same value within the error margins for both the PSS/PDADMAC and the PDADMAC/PSS/PP+ layers, meaning that both multilayers performed equally.

The retention of various salts for the PDADMAC/PSS and PDADMAC/PSS/PP+ multilayers having 5.0 and 5.5 bilayers (indicated by the arrows in Figure 7A,B) is plotted in Figure 7C. Here, the retention of NaCl in Figure 7C showed a strong odd–even effect when the PEM was terminated with either PDADMAC or PSS. The NaCl retention was highest when the multilayer was PDADMAC-terminated due to the high amount of positive charge [41]. This was also observed for the high MgCl_2_ retention and the subsequent low Na_2_SO_4_ retention for a PDADMAC-terminated PEM. When the PSS/PDADMAC PEM was PSS-terminated, the NaCl retention was low (around 10%) and an opposite Donnan-exclusion effect was observed due to the high Na_2_SO_4_ retention and relatively low MgCl_2_ retention. The same Donnan-exclusion can be seen for the PPMs independent of the terminated layer, indicating that the PPM contained a lot of negative charge. The same was seen for the PAH/PSS/PP+ PPM, most likely due to PSS dominating the charge balance within the PPM, whereas PDADMAC dominated the charge within the PEM [42]. The same conclusion holds for PSS/PAH multilayers, whereby PP+ had a lower charge density than PDADMAC, and, for this reason, when PP+ were used, the charge balance could be changed resulting in negatively charged multilayers.

### 3.3. Literature Comparison

In the previous sections, it was shown that defect–free PPMs could be fabricated using PSS as a polyanion and PP+ as the positively charged species, creating PAH/PSS/PP+ and PDADMAC/PSS/PP+ multilayer membranes. PPM-based membranes show very relevant performance as NF membranes, and it was shown that, although the PPMs did not outperform the PEMs, the charge of the multilayer could be changed. When using PP+ instead of a polycation, the overall charge of the multilayer could be altered to negative instead of positive. In this section of the article, the PPMs fabricated in this study are compared to aquaporin-containing proteoliposome- or proteopolymersome-based membranes reported in literature.

In Table 1, the permeability, salt retention, and fabrication methods of membranes fabricated in this study and values reported for aquaporin-based membranes (ABMs) in the literature are summarized. In the literature, various approaches were used in order to incorporate the vesicles. Here, most studies fixed proteoliposomes using interfacial polymerization (IP) (ABM1 [20], ABM2 [25], ABM3 [43], ABM4 [22], ABM5 [44], and ABM6 [45]). Other approaches incorporating vesicles included fixating proteoliposomes using PE cross-linking (ABM7) [46], surface imprinting polymerization with proteopolymersome (ABM8) [47], and embedding proteoliposomes into a layer-by-layer assembled membrane (ABM9) [24]. Studies fabricating supported lipid bilayers used techniques to fuse proteoliposomes on existing membranes in order to construct a supported lipid bilayer (SLB) membrane (ABM10) [48], rupture and cross-linking of proteoliposomes on a polydopamine support layer (ABM11) [49], and ruptured proteoliposomes on top of a PEM membrane (ABM12) [50]. 

ABM1–6 are membranes that incorporated proteoliposomes using IP; these membranes generally had a high NaCl retention, which is typical for IP-based membranes. For this reason, these membranes outperformed the PPM and PEM membranes fabricated in this study in terms of salt rejection. Only MgCl_2_ retention was measured for ABM7, 8, and 9, which were constructed using different incorporation methods. Here, it can be observed that the membrane performance depended hugely on the incorporation approach, where, e.g., high permeabilities of 36.6 L∙m^−2^∙h^−1^∙bar^−1^ and 85% MgCl_2_ retention were measured for ABM7, whereas, for ABM8, a high permeability of 23 L∙m^−2^∙h^−1^∙bar^−1^ was also measured but a much lower MgCl_2_ retention of 51% was obtained. ABM9 had a permeability of 6 L∙m^−2^∙h^−1^∙bar^−1^ and an MgCl_2_ retention of 95%, whereby it used PAH and PSS–PAA to incorporate proteoliposomes in the active layer using flat-sheet PAN supports. In our study, the preparation method was different, whereby PP+ were used and a multilayer was built by alternately adsorbing PP+ and polyanions, while, in the study of Sun et al. [24], only one layer of proteoliposomes was adsorbed onto the PE layer. The PAH/PSS/PP+ membranes in this study had a higher permeability of 8.7 L∙m^−2^∙h^−1^∙bar^−1^ in comparison to ABM9, but a lower MgCl_2_ retention of 50%–60% due to the negatively charged nature of this PPM. For the SLB membranes (ABM10, 11, and 12), there was quite a large spread in performance, whereby ABM11 and 12 performed well with MgCl_2_ retentions of 90% and 97%. However, ABM10 only had an NaCl retention of 20%, indicating that such layers are quickly prone to forming defects. Comparing the retention of all membranes is a difficult task, since it depends on many factors, including the surface charge and type of salt retention measured, among others. The studies in the literature mostly reported the retention of just one type of salt. For example, for ABM7, 8, 9, 11, and 12, only the retention of MgCl_2_ was measured; when membranes have a strong positive charge, MgCl_2_ retention will be high. To compare it to this study, the retention of Na_2_SO_4_ for the PAH/PSS/PP+ and PDADMAC/PSS/PP+ membranes fabricated was very high with values of 85%–90% due to the dominant negative charge of the membranes, resulting in especially high retention toward negative divalent ions instead of positive divalent ions. Such a diversity in retention behavior adds to the scope of possible applications for PEM- and PP+-based membranes. 

## 4. Conclusions

In this study, we showed that the incorporation of aquaporin-containing proteopolymersomes into PEM films is a simple and straightforward procedure. It was shown by reflectometry that a multilayer can be built using a polyanion and PMOXA–PDMS–PMOXA-based proteopolymersomes (PP+) as the positively charged species. When the two were deposited alternately, a PP+ multilayer (PPM) was fabricated, as shown here for both PAA/PP+ and PSS/PP+. These multilayers can simply be constructed on the inside of hollow fiber ultrafiltration supports to form PPM-based nanofiltration (NF) membranes. Using SEM, it was shown that PP+ were present in the PSS-based PPMs, but formed a dewetted structure for the PAA-based PPMs. When comparing the membrane performance of the PPMs, it was seen that the PAA-based PPM membranes contained defects, while the PSS-based PPM formed a good membrane with 80%–83% MgSO_4_ retention. MWCO measurements (<350 Da) further demonstrated that the PSS-based PPM membranes formed very relevant NF membranes. In comparison to PEM membranes (PAA/PAH, PSS/PAH, and PSS/PDADMAC), no performance increase in either selectivity or permeability was observed. However, dominant negatively charged layers could be formed when using PPMs instead of the dominant positively charged PEMs. This was most likely due to the relative low charge density of the PP+ in comparison to PSS. When the PPM membranes were compared to the literature, IP-based aquaporin-containing membranes outperformed the membranes in this study based on salt rejection. However, our membranes showed similar performances in terms of permeability and retention with non-IP-based aquaporin-based membranes, while being more optimized toward the retention of divalent anions. In summary, this study provides a fundamental understanding of the build-up of PPM membranes and the resulting membrane performances, and it shows that well-performing hollow fiber-based PPM membranes can be fabricated using a very simple approach.

## Figures and Tables

**Table 1:** Summarized values for permeability, salt retention, and incorporation methods for aquaporin-based membranes (ABMs) in this work and the literature, as well as PEM membranes in this study. N/A—not applicable.

Membrane Type	Incorporation Approach	Permeability (L∙m^−2^∙h^−1^∙bar^−1^)	Salt Concentration	Salt Retention
Polyelectrolyte multi-layer (PEM)				
[PSS/PDADMAC]_5_PSS	N/A	5.4	5 mM	9% NaCl, 67% MgCl_2_ 90% Na_2_SO_4_, 89% MgSO_4_
[PSS/PAH]_5_PSS	N/A	12.3	5 mM	26% NaCl, 96% MgCl_2_ 80% Na_2_SO_4_, 94% MgSO_4_
[PAA/PAH]_5_PAA	N/A	2.6	5 mM	9% NaCl, 57% MgCl_2_ 34% Na_2_SO_4_, 60% MgSO_4_
Proteopolymersome multilayer (PPM)				
PDADMAC[PSS/PP+]_4_PSS	LbL^a^	3.5	5 mM	8% NaCl, 48% MgCl_2_ 90% Na_2_SO_4_, 84% MgSO_4_
PAH[PSS/PP+]_4_PSS	LbL^a^	7.7	5 mM	11% NaCl, 54% MgCl_2_ 85% Na_2_SO_4_, 80% MgSO_4_
PAH[PAA/PP+]_4_PAA	LbL^a^	0.6	5 mM	8% NaCl, 17% MgCl_2_ 1.0% Na_2_SO_4_, 10% MgSO_4_
Literature values				
ABM1 [20]	IP^b^	4.0	10 mM	97% NaCl
ABM2 [25]	IP^b^	8.0	500 ppm	97.5% NaCl
ABM3 [43]	IP^b^	3.2	1000 ppm	69.6% MgSO_4_, 16.5% NaCl
ABM4 [22]	IP^b^	6.4	500 ppm	93.5% NaCl
ABM5 [44]	IP^b^	1.3	2000 mg/L	99.1% NaCl
ABM6 [45]	IP^b^	2.4	2000 ppm	99.6% NaCl
ABM5 [46]	CL^c^	36.6	100 ppm	85% MgCl_2_
ABM6 [47]	Pol^d^	23	200 ppm	51% MgCl_2_
ABM7 [24]	LbL^a^	6.0	200 ppm	95% MgCl_2_
ABM8 [48]	SLB^e^	4.8	1 mM	20% NaCl
ABM9 [49]	SLB^e^	6.3	2000 ppm	90% MgCl_2_
ABM10 [50]	SLB^e^	5.5	0.5 g/L	97% MgCl_2_

**^a^** Layer-by-layer assembly. ^b^ Interfacial polymerization. ^c^ Cross-linking. ^d^ Polymerization. ^e^ Supported lipid bilayer.

## Supplementary Materials

The following are available online at https://www.mdpi.com/2077-0375/10/5/103/s1, Figure S1: transmission electron microscope image of a PP+ sample.
