# Supplementary material for: Aquaporin-Containing Proteopolymersomes in Polyelectrolyte Multilayer Membranes
Source: Membranes (Basel). 2020 May 18;10(5):103. doi: 10.3390/membranes10050103 (PMC7281279; doi:10.3390/membranes10050103)
Supplement: Supplementary file 1 [file membranes-10-00103-s001.pdf]

Supplementary Information

Aquaporin Containing Proteopolymersomes in Polyelectrolyte Multilayer Membranes

Dennis M. Reurink,[a] Fei Du,[a] R. Górecki,[b,c] Hendrik D.W. Roesink,[a] Wiebe M. de Vos[a,*]

[a] Membrane Science & Technology, University of Twente, MESA+ Institute for Nanotechnology, P.O. Box 217, 7500 AE Enschede, The Netherlands

[b] Department of Environmental Engineering, Technical University of Denmark, Bygningstorvet 115, 2800, Kongens Lyngby, Denmark

[c] Aquaporin A/S, Nymøllevej 78, 2800, Kongens Lyngby, Denmark

* Corresponding author: w.m.devos@utwente.nl

TRANSMISSION ELECTRON MICROSCOPY

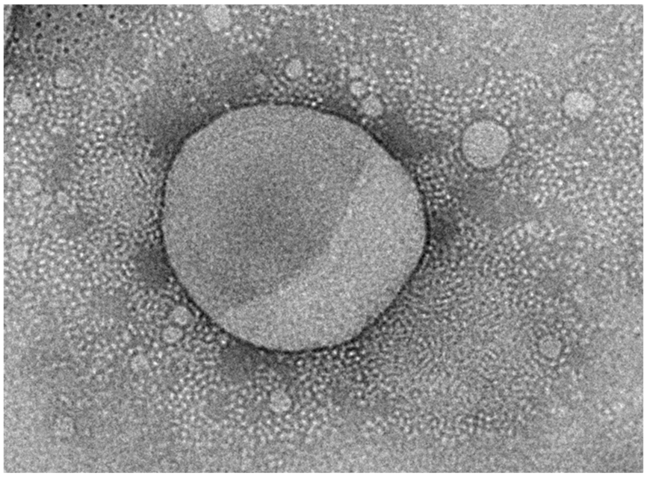

Figure S1. Transmission electron microscope image of a PP+ sample.
